# Periscope

**Published:** 1882-01

**Authors:** 


### PERISCOPE.

North American Journal of Homœopathy, August:—Dr. Straube writes upon "*Malandrium* in Small Pox,'' giving some partial provings and twenty-six clinical cases treated with it. The following symptoms are reported cured with *Malandrium*: Aching in limbs; headache; pains in left side of head with great debility; pains in back, head and abdomen; pain in head and back, fatigue, chilliness and vomiting; stiff neck; constipation; inability to go up stairs from weakness; lazy, weak feeling; terrible itching after an attack of small pox; foul smelling diarrhœa, chilliness, weak, tired feeling; great pain around the navel, sore aching in bones, sleeplessness during an attack of small pox. Crying and ill temper of children during small pox; pain in back extending to heels, soreness of abdomen, every bone in body aches. There was no smell from the eruption after *Malandrium* was given.

"S. L." translates from "Hygea" the following: Angina involving pharynx, tonsils, uvula and velum; parts inflamed and red; constant desire to swallow, with great painfulness. *Lachesis* 29 cured in three days. In another case there were copious secretions of mucus and foul breath, *swallowing almost impossible, everything being returned* through the nose. *Lachesis* 30 cured in seven days without suppuration setting in.

A healthy man had *frequent urging to urinate, with severe cutting and burning pains in the urethra; urine scanty; urine highly charged with mucus.* These symptoms were relieved by *Sulphur* in twenty-four hours, and cured in three weeks. *Cannabis, Cantharis* and other remedies had been given without effect.

A woman who had constant urging to urinate, with pain during discharge; and after urinating, drawing pain along ureters, compelling her to bend double, was cured with *Sulphur.*

Three ladies had cutting pain in umbilical region extending to ovaries; urging to urinate, with scanty discharge of turbid mucous urine, having a mucous deposit. Labor-like pain in course of ureters, extending to the thighs, with burning in the urethra. During stool some tenesmus and cutting. *Colocynthis* cured.

A boy suffered from excruciating attacks of headache, during which he swayed the head to and fro, or pressed it strongly against some hard object. After the attack, desire for something to eat. *Arsenicum* 12, one dose cured. A woman who had the same symptoms received the same remedy and was similarly cured.

American Homœopath, August:—Dr. Danforth reports a a case of a woman who had severe pains in left temple, cardiac and left ovarian region. Principal symptoms: *great mental depression, aversion to talking; sensation as if heart and ovary were drawn up together;* a sense of *contraction* or *drawing together* between the organs. Cured with *Najar tripujians.*

Dr. Brace was called to a case of angina pectoris. Patient suffering much agony; *greatly prostrated, surface cold.* *Arsenicum* 3, one dose cured.

Dr. Clarke had a case of poisoning from arsenic by working with it in bird stuffing. Also by breathing it from the colored paper on the walls. *Carbo. veg.* 6 cured.

Dr. Burnett had a case of catarrhal stenosis of lachrymal duct in a baby, a few months old. The eye was constantly watering. The old school doctor called it "a cold." Dr. Burnett being called in gave *Natrum mur.* 6 and cured it. The remedy was observed to cause vomiting.

Dr. Brazie reports a case of helminthiasis, exhibiting chills, convulsions and fever every day for six days; *diarrhœa of white stools, turbid urine, great hunger. Cina* 200 cured in three days.

Medical Counselor, August.—Dr. Brigham reports the following: A child had bilious intermittent fever. Chill at 8 A. M. *More shivering than the degree of cold warrants. During chill, thirst, headache, nausea and vomiting of bilious matter. Eupatorium* cured.

A boy had diarrhœa, *stools at* 3 *o'clock,* A. M. Cured with *Kali carb*. A man had army diarrhœa which seemed incurable. *Diarrhœa always worse in summer and warm weather; worse also from motion. Chills creep over* him. Cured with *Bryonia* 200.

Homœopathic Courier, August.—" K." reports a case of nasal catarrh cured with *Nat. mur.* 12 in two weeks. The only indications obtained were: *thirst and inordinate desire for salt.* A child had an eczema of scalp and face, with dry scales burning after scratching. *Calcarea* 200 cured.

A case of persistent vomiting, even water being thrown up as soon as it reached the stomach, with tenderness of pit of stomach, tongue red at tip, great thirst, fluids gurgling as they are swallowed, was relieved by *Amygdalis Persica.* To a woman suffering with consumption of the lungs, *Calcarea silicata* was given, which produced several mental symptoms, among which may be mentioned: Conviction that she is surrounded with corpses. Imagines that friends who are dead have returned. Carries on imaginary conversations with the dead. The lung symptoms were, however, relieved. As the above mental state continued, *Hyoscyamus* 2C was given, which relieved the mental state. She is now completely cured.

From the *Rundschau* is translated a case of sciatica with intense pains not controlled by the largest doses of morphia and chloral. As the pain had been excited by a fright, and as there were swollen inguinal glands and minute green spots on the linen, sycosis seems to have been suggested to the doctor as the cause. Accordingly *Thuja* was given with curative results that were "striking."

Hahnemannian Monthly, September.—Dr. Hoopes relates five cases of abnormal labor pains cured by the appropriate remedy as follows: A woman in labor, tossed about the bed *in great agony, weeping and declaring that she could endure the pains no longer.* One dose of *Coffea,* 2C removed the distress, and the labor became normal.

Another woman in labor was *so cross she would not hardly speak. Chamomilla* 2C was given; the distressing character of the pains was relieved, and the ill humor disappeared.

The pains then showed a disposition *to come and go suddenly. Belladonna* 2C made them regular. In another case *every labor pain caused urging to stool. Nux vom.* 6 relieved at once. Another patient experienced pains which extended from the hypogastrium and he hip to the thighs. *Nervous excitability; felt that she could not bear the pains any longer. Coffea* 3C in repeated doses relieved. A fifth case was nervous, and the pains ran from the hypogastrium upward. *Gelseminum* 30 corrected the pains. The doctor says he could cite *many more* such cases.

Bibliotheque Homœopathique, August.—Dr. Chancerel reports a case of scrofula in a young woman. The scalp was affected with an eruption, which was infested with lice. Upon the soles of the feet an eruption of brown pustules interfering with walking. Amenorrhœa and hæmorrhoids. *Sulphur* was administered, and in fourteen days the eruption upon the feet was almost entirely removed. Then, rheumatic symptoms occurring. *Rhus tox.* was prescribed, and in six weeks more the lice had disappeared. Several other remedies were given at intervals during the next eight months for various complications, ending finally with *Pulsatilla,* which cured. The doctor then argues that *Pulsatilla* was indicated at the first, but that he did not know it, and hence the treatment was prolonged.

A man of sixty years of age had acute cystitis from taking cold. He had frequent desire to urinate. Tenesmus of neck of bladder, Dull pain in hypogastrium and loins, and brick-dust sediment in urine. *Lycopodium* 30 cured.

Homœopathic Journal of Obstetrics, August (November number not received):—Dr. Hale reports the cure of several cases of nausea and vomiting in pregnancy with *Jaborandi 2x dilution*; the indication being profuse *salivation with nausea and vomiting.* The same remedy also cured a similar condition where instead of

*salivation*, there was *dryness of mouth; saliva like cotton; red tongue and parched lips.*

Dr. Farrington reports a case of threatened miscarriage with severe backache; *severe cramping, bearing down pains almost unbearable, causing constant restless moving about in bed. Cannot sit or lie still, so severe are the pains. Viburnum Op.* 2, in accordance with the above italicized indications, relieved at once. Later, she became frightened, and the pains, returned. *Viburnum* failed to relieve, because the patient became *irritable, nervous, and declared in a petulant way that she neither could nor would stand the pains.* These symptoms indicated *Chamomilla* 30, which was accordingly given and cured. Dr. F. draws an important lesson from the case.

Dr. P. P. Wells gives an interesting paper upon "The Power of the Specific Remedy," relating cases of displacement of the uterus, cured with medicines alone, no mechanical interference being permitted. It is much to be regretted that the doctor did not state what these medicines were. It would be of much advantage to know.

